# T-Cell Epitope-Based Vaccines: A Promising Strategy for Prevention of Infectious Diseases

**DOI:** 10.3390/vaccines12101181

**Published:** 2024-10-17

**Authors:** Xin Song, Yongfeng Li, Hongxia Wu, Huaji Qiu, Yuan Sun

**Affiliations:** State Key Laboratory for Animal Disease Control and Prevention, Harbin Veterinary Research Institute, Chinese Academy of Agricultural Sciences, Harbin 150069, China; songxinor@163.com (X.S.); liyongfeng@caas.cn (Y.L.); wuhongxia@caas.cn (H.W.)

**Keywords:** T-cell epitopes, cell-mediated immune responses, epitope identification, vaccines, infectious diseases

## Abstract

With the development of novel vaccine strategies, T-cell epitope-based vaccines have become promising prophylactic and therapeutic tools against infectious diseases that cannot be controlled via traditional vaccines. T-cell epitope-based vaccines leverage specific immunogenic peptides to elicit protective T-cell responses against infectious pathogens. Compared to traditional vaccines, they provide superior efficacy and safety, minimizing the risk of adverse side effects. In this review, we summarized and compared the prediction and identification methods of T-cell epitopes. By integrating bioinformatic prediction and experimental validation, efficient and precise screening of T-cell epitopes can be achieved. Importantly, we delved into the development approaches to diverse T-cell epitope-based vaccines, comparing their merits and demerits, as well as discussing the prevalent challenges and perspectives in their applications. This review offers fresh perspectives for the formulation of safe and efficacious epitope-based vaccines for the devastating diseases against which no vaccines are currently available.

## 1. Introduction

Vaccination is a proven, safe, and cost-effective way to protect against infectious diseases [1,2]. There is an urgent need for safe and effective vaccines against some devastating diseases, including African swine fever (ASF), acquired immune deficiency syndrome (AIDS), and porcine reproductive and respiratory syndrome (PRRS). Antibodies are very important for the efficacy of vaccines, but there is mounting evidence indicating that cell-mediated responses are associated with early-onset and long-lasting protection conferred by immune memory, even in the absence of humoral immune responses. Cell-mediated immune responses, an essential component of immune defense, have evolved to recognize and control intracellular pathogens [3]. Cell-mediated immune responses are far less affected by viral variants [4]. Moreover, although neutralizing antibodies generally diminish more swiftly than immune responses mediated by cells, enduring T-cells usually inhibit severe viral infections by directly destroying infected cells or assisting other immune components. Notably, T-cells exhibit greater cross-reactivity compared to antibodies. Consequently, diverse pathogens are unlikely to evade long-lasting, broadly cross-reactive immune responses driven by T-cells [2,5,6]. Most current vaccines provide predominantly antibody-based protection and are limited in their ability to induce the cell-mediated immune responses that are able to protect against genetically diverse viruses and/or protect from severe disease [2]. Consequently, vaccines developed in the future that aim to enhance both the functions of T-cell effectors and antibody responses might offer improved protection [2].

T-cell epitope-based vaccines target specific immunogenic peptide sequences to elicit protective T-cell responses against pathogens. In contrast to traditional vaccines, which primarily elicit a humoral immune response, these innovative vaccines are engineered to activate a precise cell-mediated immune response. They are strategically designed to focus on the highly conserved regions of the virus, which are less susceptible to mutation. This approach aims to provide enduring protection and mitigates the potential for the virus to evade the immune system. In contrast, traditional vaccines may be less effective in the face of viral mutations and typically involve a more complex and costly production process, as well as a higher risk of side effects. Consequently, T-cell epitope-based vaccines present a promising strategy for combating rapidly mutating pathogens, while traditional vaccines remain valuable for inducing broad immune responses [1,2]. Over the past few decades, T-cell epitope-based vaccines have achieved significant strides. In the 1980s and 1990s, the concept of T-cell epitopes gained widespread acceptance, and researchers began exploring their use in vaccine design [7,8,9]. In the 2000s, advancements in genomics, proteomics, and immunoinformatics enabled more accurate and rapid prediction of T-cell epitopes and vaccine design [10]. Since 2010s, bioinformatic tools have been employed to identify T-cell epitopes and design vaccines against vaccinia and variola [11,12]. With the outbreak of the COVID-19 pandemic in 2020, research on T-cell epitope-based vaccines accelerated, with studies using immunoinformatics methods to predict the T-cell and B-cell epitopes of SARS-CoV-2, providing a foundation for vaccine development. Furthermore, the advent of messenger RNA (mRNA) vaccine technologies has facilitated the development of vaccines that are based on T-cell epitopes [13].

Generally, the epitopes recognized by T-cells are short peptides derived from the processing of antigens found in both structural (components of the viral particles) and non-structural proteins (e.g., enzymes and transcription factors) of viruses [14,15]. These epitopes are the main components in the development of epitope-based vaccines and immunotherapies for viral infections, tumors, and autoimmune diseases [16,17,18]. T-cell epitope-based vaccines have been preliminarily confirmed to be safe and effective [19,20,21]. Antigen processing and presentation refer to the process where antigen-presenting cells (APCs) in the immune system process foreign or self-antigens into short peptides and present them to T cells via surface molecules, thereby activating T cell-mediated immune responses [22]. Peptides presented by the major histocompatibility complex (MHC) serve as crucial molecular structures that are recognized by both T-cell receptors (TCRs) and B-cell receptors (BCRs), as well as soluble antibodies. This recognition triggers cell-mediated immune responses, underscoring their importance in the immune system.

In this review, we focus on the synthesis and current challenges for T-cell epitope-based vaccines, as well as epitope identification methodologies, aiming to provide a reference for the design and development of vaccines.

## 2. T-Cell Epitope Mapping for Vaccine Development

### 2.1. In Silico Prediction of T-Cell Epitopes

T-cells scan MHC ligands on nucleated cells (MHC-I) and APCs or lymphoid cells (MHC-I/-II) to identify antigens from pathogens and cancer cells. The identification of epitopes serves as the initial crucial step in constructing epitope-based vaccines, and numerous methods have been devised for this purpose. Immunoinformatics methods have been employed to predict epitopes and analyze their physicochemical properties, reactivity, and immunogenicity [23,24,25]. We summarized the methods used in the prediction and analysis of T-cell epitopes in Table 1.

**Figure 1:**
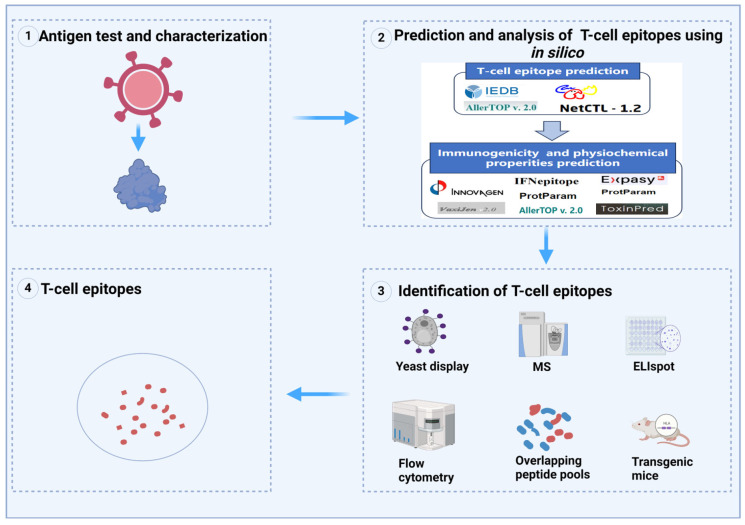
T-cell epitope mapping [54,55,56,57]. (**1**) Immunogens are selected as screen targets; (**2**) T-cell epitopes are predicted and tested using various bioinformatic methods; (**3**) T-cell epitopes are identified by various experimental methods; (**4**) Acquisition of candidate epitopes.

The following process can serve as a reference for epitope prediction (Figure 1). The Immune Epitope Database (IEDB) serves as a widely used online tool for predicting T-cell epitopes. It provides a repository of experimental data on antibody and T-cell epitopes across various domains, including infectious disease, allergy, autoimmunity, and transplantation [44]. The updated IEDB-3D 2.0 comprises a complete overhaul of how we obtain and present 3D structural data [44]. By encompassing information on virus strains and species, it facilitates the derivation of final scores for specific peptide-MHC complexes (pMHCs) [45]. The widely utilized SYFPEITHI score (a publicly accessible database for MHC ligands and peptide motifs was established, and it was named SYFPEITHI in honor of the first peptide sequenced directly from an MHC eluate) is derived from ligand elution data analysis and average relative binding matrices, which rely on measured binding data from single substitution analogs of known ligands [46,47]. An increasing number of bioinformatic methods are continuously enhancing analysis systems by collecting clinical data, enabling algorithms to make more accurate predictions in the future. Once an epitope peptide is identified, it is crucial to thoroughly analyze its immunogenicity and physiochemical properties, such as its hydrophobicity. Several studies have demonstrated that hydrophobic peptides tend to exhibit more immunogenicity, making them promising candidates for use as effective epitopes [48]. However, it is important to note that the synthesis of these hydrophobic peptides can present challenges due to their unique chemical properties [20,48]. These epitopes can be comprehensively analyzed by the Vaxijen 2.0 server, which provides immunogenic profiling based on the physicochemical properties of the proteins. Additionally, to ensure the safety of potential therapeutic applications, the AlgPred v2.0 (Indraprastha Institute of Information Technology, New Delhi, India, https://webs.iiitd.edu.in/raghava/algpred2 (accessed on 20 July 2021)) and ToxinPred 3.0 server (Indraprastha Institute of Information Technology, New Delhi, India, https://webs.iiitd.edu.in/raghava/toxinpred3/ (accessed on 21 July 2024)) programs are utilized to assess the allergenicity and toxicity of these epitopes, respectively [49,50]. Certainly, numerous software options are available to facilitate these data analyses (Table 1).

Despite their widespread utilization in predicting epitopes, bioinformatic methods possess several limitations. Antigen processing and presentation involve protein degradation, peptide cleavage, transport to the endoplasmic reticulum, and binding to MHC. Additionally, the predictive models may not account for the manipulation of cellular processes by viruses, which can impact antigen presentation, nor could they capture the dynamic changes in viral protein expression during the infection [15]. Different MHC subtypes display various peptides, and the distribution abundance of MHC subtypes varies among several subspecies within the same species [51]. Additionally, there is a notable scarcity of analytical structures for the TCR–MHC complex in animal models. It is also important to note that T-cell epitopes vary between species, and the surface markers differ accordingly, influencing the target antigens of T cells retained after central tolerance. For example, some viruses can cause severe immune reactions in humans, but not in poultry. Therefore, epitope prediction must be tailored to specific species [52]. Certainly, it is important to acknowledge that while the evaluation of the calculation methods may not yield completely accurate results, they still provide valuable insights for experimental verification [53]. To overcome these challenges, it is imperative to accelerate the advanced development of artificial intelligence (AI) technologies. This involves leveraging extensive experimental datasets to thoroughly train AI models, enabling them to effectively navigate and respond to a wide range of complex scenarios.

### 2.2. Approaches to the Validation of T-Cell Epitopes

After epitopes are predicted using the aforementioned methods, additional validation is necessary. Epitope identification is highly contingent on several context-dependent variables, including host, source organism, and immune reaction. T-cell epitopes mapping technology mainly includes the overlapping of a peptide library [58], pMHC isolation and mass spectrometry (MS) [15,19], phage display [59], proliferation assay [60], tetramer staining [12], flow cytometry analysis [61], enzyme-linked immunosorbent spot assay (ELIspot) [62], the development of a cell-free antigen processing system, liquid chromatography-tandem mass spectrometry (LC-MS/MS) [63], the synthesis of MHC multimers [64], and the generation of a transgenic mice expressing HLA [65]. Numerous studies have yielded crucial insights into the development of innovative epitope-based vaccines, which precisely target pathogenic antigens by identifying antibody epitopes generated post-vaccination. For example, this strategy highlights the importance of blocking human monoclonal antibody epitopes in malaria vaccines by elucidating the mechanisms by which these vaccines elicit immune responses against specific pathogens [66]. A major challenge in identifying T-cell epitopes lies in their considerable variability in recognition among individuals affected by genetic and environmental factors [17]. Therefore, utilizing multiple methods in parallel can enhance the accuracy and reliability of epitope identification (Figure 1).

## 3. Novel Approaches to the Development of T-Cell Epitope-Based Vaccines

**Figure 2:**
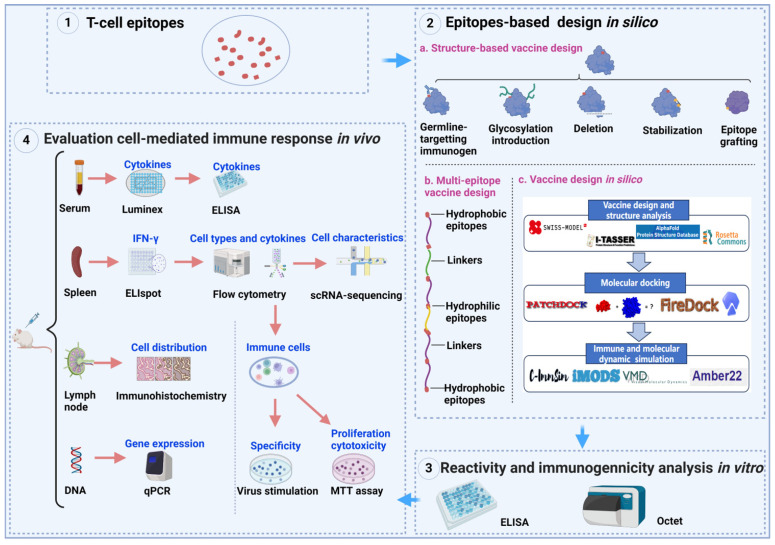
A well-established workflow for the rational design of T-cell epitope-based vaccines [1,54,92,93]. (**1**) Mapping of T-cell epitopes based on Figure 1. (**2**) *In silico* epitope-based vaccine design encompasses various approaches, including structure-based and multi-epitope-based vaccine design: (**a**) Epitope-based vaccine design involves fully displaying dominant antigen epitopes based on protein structure. Design strategies include germline-targeting immunogen, glycosylation introduction, deletion, stabilization, and epitope grafting. (**b**) Multi-epitope-based vaccine design integrates the physicochemical properties of epitopes, arranging them based on their characteristics and utilizing different linkers to connect epitopes in sequence. (**c**) Vaccine design *in silico* leverages various AI technologies. (**3**) Upon vaccine development and synthesis, reactivity and immunogenicity are analyzed *in vitro* using ELISA and Octet. (**4**) Vaccine candidates identified through the aforementioned steps are subsequently subjected to *in vivo* immune assessments to evaluate their efficacy. Evaluation methods include ELIspot, flow cytometry, single-cell RNA sequencing, Luminex, ELISA, immunohistochemistry, and qPCR.

Novel vaccines based on T-cell epitopes offer innovative solutions for infectious diseases that are difficult to control effectively with traditional vaccines, such as inactivated vaccines. Additionally, the conservation of T-cell epitopes underscores the potential of epitope-based vaccines as a scientifically sound and precise approach to future vaccine development. Epitopes identified via rigorous screening and selection can be useful in developing diverse epitope-based vaccines (Table 2). Here, we discuss some novel strategies to develop epitope-based vaccines (Figure 2).

**Table 2:** Summary of recently developed T-cell epitope-based vaccines.

Types	Pathogens	Cell-Mediated Immune Responses	Efficacy	Clinical Issues	Phases	References
Subunit vaccine	RSV	/	Protective	It may cause neurological disorders	Licensed	[67,68]
	Influenza virus	CD4^+^ T cells	Persistent cell-mediated immune response	Failed to improve antibody responses	Phase II	[21]
	SARS-CoV-2	IFN-*γ*, IL-2, CD4^+^/CD8^+^ T cells	Strong, long-term, and broad-spectrum immune responses	Mild and transient injection site pain and fatigue	Phase I/II	[69]
	*Mycobacterium tuberculosis*	T helper 1 (Th1) cells, CD4^+^ T cells	Highly immunogenic	The duration of the immune responses was not evaluated	Preclinical (in mice)	[70]
	Zika virus	CD4^+^/CD8^+^ T cells	Highly immunogenic	The neutralizing antibodies were not elevated significantly	Preclinical (in mice)	[71]
	HIV	/	Broad neutralizing antibodies	The duration of the immune responses was not assessed	Preclinical (in guinea pigs and rhesus macaques)	[72]
	EBV	CD4^+^/CD8^+^ T cells	Protective	Further clinical trials are needed	Preclinical (in mice)	[73]
mRNA	Human metapneumovirus	/	Potential candidates	Further *in vivo* models are needed	Preclinical (without animal experiment)	[43]
	SARS-CoV-2	/	Potential candidates	Further model validation is warranted	Preclinical (without animal experiment)	[74]
	*Pseudomonas aeruginosa*	/	Potential candidates	Further laboratory and clinical trials are required	Preclinical (without animal experiment)	[75]
	Influenza virus	/	Potential candidates	Further laboratory and clinical trials are required	Preclinical (without animal experiment)	[76]
	Rotavirus	IFN-*γ*/TNF double-positive CD8^+^ and CD4^+^ T cell responses	Highly immunogenic	Humanized animal models are needed	Preclinical (in rodents)	[77]
Vector vaccine	SARS-CoV-2	/	Protective	Humanized animal models are needed	Preclinical (in hamster)	[78]
	SARS-CoV-2	Memory T cells	Protective	The duration of the immune responses was not evaluated	Preclinical (in mice)	[79]
	Infectious bronchitis virus (IBV)	IFN-*γ*	Protective	Further clinical trials are required	Preclinical (in chickens)	[80]
Cocktail	Cancer	CD4^+^/CD8^+^ T cells	Strong T-cell responses	More clinical trials are required	Phase I	[81]
Biopolymer particles	Influenza virus	CD8^+^ T cells	Protective	Further clinical trials are required	Preclinical (in mice)	[82]
Virus-like particles (VLPs)	SARS-CoV-2	Memory CD8^+^ T cells, IFN-*γ*	Humoral and cell-mediated immune responses	Non-protective against SARS-CoV-2	Preclinical (in mice)	[83]
DNA vaccine	Human leishmaniasis	CD4^+^/CD8^+^ T cells	Protective	Further clinical trials are required	Preclinical (in rodents)	[84]
DC vaccine	SARS-CoV-2	CD8^+^ T cells	Effective against SARS-CoV-2	Further clinical trials are required	Preclinical (in mice)	[85]
Exosomal Vaccine	SARS-CoV-2	CD8^+^ T cells	Robust cell immune responses	Further clinical trials are required	Preclinical (in mice)	[86]
Reverse vaccinology	Ebola virus	/	Potential candidates	Further model clinical trials are required	Preclinical (without animal experiment)	[87]
Ghost vaccine	Avian influenza virus and Newcastle disease virus (NDV)	/	Potential candidates	Further model clinical trials are required	Preclinical (without animal experiment)	[88]
Nanoparticle	Influenza virus	CD4^+^/CD8^+^ T cells	Protective	Further model clinical trials are required	Preclinical (in mice)	[89]
	Influenza virus	/	Less clinical signs	The generalizability of the human challenge model	Phase IIb	[90]
	SARS-CoV-2	IL-4 and IFN-*γ*, CD4^+^/CD8^+^ T cells	Protective	Further model clinical trials are required	Preclinical (in mice)	[91]

### 3.1. T-Cell Epitope-Based Subunit Vaccines

Several studies have confirmed the effectiveness of subunit vaccines as platforms for delivering T-cell epitope-based vaccines. Instances encompass viruses with significant antigenic variability, such as HIV and influenza. Broadly neutralizing antibodies have been identified and structurally characterized alongside their corresponding target epitopes for these viral pathogens [1,67,94]. However, neutralizing epitopes are usually small in size and thus weak in immunogenicity. Therefore, the rational design of epitope-based vaccines is critically important, aiming to enhance these aspects by focusing on well-defined conformational epitopes.

One of the promising strategies to enhance immunogenicity is to design vaccines based on protein structure. It is worth noting that relatively short linear T-cell epitopes often lack a well-defined structure, which can lead to limited immunogenicity and stability *in vivo*. The goal of structure-based design is to enhance the immunogenicity, yield, and stability of immunogens. Initially, the primary objective of vaccine design was to elucidate the protein structure of immunogens. Understanding the molecular structures of these immunogens enables precise design to elicit targeted immune responses against pathogens. Traditionally, molecular design analysis was primarily and reliably applied to protein structures determined by X-ray crystallography, which offers heightened sensitivity to atomic details. Additionally, techniques like nuclear magnetic resonance (NMR) or cryo-electron microscopy (cryo-EM) have also been used to determine high-resolution three-dimensional (3D) structures of target antigens [1]. Conventional techniques for detailed atomic-level protein design were primarily applicable to structures determined by the X-ray crystallography, due to the strong reliance of design processes on accurate atomic data [95]. However, recent advancements in AI-driven structure prediction methods, including AlphaFold3 [96], have shown remarkable precision that competes with the accuracy typically associated with crystallographic techniques. The basic principles of epitope-based design include eliminating invalid epitopes to achieve antigen accuracy and a focus on stable, fully exposed antigen epitopes. DS-Cav1 serves as a successful example of this approach [68]. Structure-based design yielded stabilized versions of the respiratory syncytial virus (RSV) F protein that maintained antigenic site Ø under extreme conditions of pH, osmolality, and temperature. Six crystal structures of the RSV F protein offered atomic-scale insights into the ways in which added cysteine residues and occupied hydrophobic cavities enhanced stability. When mice and macaques were immunized with the site Ø-stabilized variants of the RSV F protein, they elicited RSV-specific neutralizing antibody levels that significantly surpassed the protective threshold [68]. Recent advancements have refined this approach, utilizing strategies such as disulfide bond (Schrodinger BioLuminate, New York, NY, USA), cavity-filling (Rosetta), and electrostatic charge (MOE v2014, Rosetta) [67]. These efforts have culminated in the successful market launch of the vaccine. Recent developments in the fields of protein design and engineering have utilized strategies that merge calculations based on structure and sequence with machine learning techniques, greatly improving the optimization of proteins and facilitating the novel design of their structure and function [94]. The evolution of AI-driven protein design has shifted from approaches that maintain a fixed backbone to more adaptable methods that rely on predictions of structure derived from sequences, as well as algorithms that generate both structure and sequence [97]. Numerous natural proteins demonstrate poor stability, characterized by minimal energy differences that separate the native state from various unfolded or misfolded forms, presenting a significant challenge within protein engineering [98]. Efforts to address this challenge include the development of thermally and conformationally stable protein scaffolds that accurately mimic viral neutralization epitopes, effectively inducing neutralizing activity in vaccinated macaques [1]. Stability-focused design methods have also proven effective in enhancing the yield of functional proteins. For instance, an engineered variant of the SARS-CoV-2 spike protein demonstrated a tenfold increase in production levels in mammalian cells, highlighting significant improvements in expression efficiency without compromising protein activity [99].

To design a multi-epitope-based vaccine construct, selected epitopes are linked using specific linkers to enhance immunogenicity while minimizing concerns associated with carriers. This approach is sometimes referred to as “string-of-beads vaccines” [100]. Each epitope’s amino acid sequence undergoes hydrophobicity analysis via the ProtParam tool to guide arrangement, positioning relatively hydrophobic epitopes centrally and hydrophilic ones at the ends. Furthermore, the ordered epitopes are connected by linkers (GPPGPG, LRMKLPKS, AAYKK, and EAAAK) [101]. Moreover, the vaccine’s interaction with immune receptors is evaluated through docking studies to assess its ability to stimulate immune responses. Dynamic simulations further confirm strong binding potential with immune receptors, indicating robust potential for both humoral and cell-mediated immune responses [43]. For instance, the Multimeric-001 (M-001), a synthetic peptide vaccine derived from conserved immunogenic epitopes from influenza type A and B strains (BiondVax Pharmaceuticals, Jerusalem, Israel, US NCT: 02691130) [21] illustrates this approach. Therefore, the rational selection and arrangement of epitopes are critical in constructing effective multi-epitope vaccines. Bioinformatic tools significantly streamline vaccine design by simulating constructs and analyzing their physicochemical properties *in silico*, reducing the experimental workload.

### 3.2. T-Cell Epitope-Based mRNA Vaccines

mRNA vaccines can induce cell-mediated immune response by expressing the T-cell epitopes of the target antigens, as demonstrated in SARS-CoV-2 [73] and rotavirus [77]. For example, a monomeric P2-VP8* mRNA vaccine candidate is being developed as a rotavirus vaccine targeting its VP8 spike protein. Proteins isolated from vaccine candidate LS-P2-VP8* have been shown to be secreted *in vitro* following self-assembly into nanoparticles that display VP8* [77]. Firstly, virus-specific proteins and epitopes are selected. The mRNA sequences are then engineered to encode these epitopes, with optimization of non-coding regions to enhance their immunogenicity. This formation aims to improve vaccine stability, immune cell uptake, and overall efficacy in stimulating a robust immune response. Throughout this process, rigorous testing and refinement ensure that the mRNA vaccine constructs effectively express the chosen epitopes and elicit targeted immune responses against the virus. This approach harnesses modern biotechnological advancements for the development of vaccines tailored to specific viruses, leveraging the benefits of mRNA technology, such as rapid development, scalability, and potentially enhanced immunogenicity. The mRNA vaccine platform has shown potential for future vaccines, although most epitope-based mRNA vaccines are still in preclinical stages [43,74,76]. Consequently, because of the numerous benefits, such as time efficiency, safety, and effectiveness, the advancement of mRNA vaccines ushers in a new age in the field of vaccinology. Additionally, the potential for mRNA vaccines, which has been tested through bioinformatics, certainly necessitates experimental validation to confirm its efficacy and safety *in vivo*.

### 3.3. T-Cell Epitope-Based Vector Vaccines

This method harnesses the replication and expression capability of viruses to stimulate the T-cell immune response by inserting the T-cell epitope of the pathogen into the virus/bacteria vector. Tan et al. developed a recombinant thermostable NDV vaccine that expresses IBV multiple epitopes, offering protection against either IBV or NDV challenge [102]. PanCoVac is a candidate vaccine containing multiple conserved T-cell epitopes from the coronaviral structural proteins delivered by a non-integrating lentivirus vector (Institute of Virology, Charité-Universitätsmedizin Berlin, Berlin, Germany). In animals, a single low-dose intranasal administration of PanCoVac followed by infection with the ancestral SARS-CoV-2 resulted in asymptomatic outcomes and a significant reduction in lung viral loads. This vaccine holds promise for protection against severe diseases caused by SARS-CoV-2 variants and emerging pathogenic coronaviruses [78]. Additionally, a COVID-19 vaccine utilizing a chimpanzee adenoviral vector, which includes a range of immunogens, a modified full-length spike protein, and conserved T-cell epitopes derived from SARS-CoV-2 [79]. In the design of viral vector vaccines, the pre-existing antibodies against the virus vector need to be considered. For instance, many individuals may have immunity to certain adenoviruses, potentially reducing the vaccine’s efficacy. Therefore, assessing and addressing this challenge is critical to enhancing vaccine efficacy. While adenovirus vectors are currently the most widely used vaccine delivery system, it is expected that more suitable vectors will be developed in the future. Of course, it is essential to consider various factors, including the different hosts and strains of vectors present in viruses or bacteria.

### 3.4. Other Delivery Platforms and Adjuvants to Enhance the Immunogenicity of T-Cell Epitope-Based Vaccines

With technological advancements, numerous delivery platforms have emerged, including exosome-mediated delivery, water-in-oil nanoemulsion, lipid nanoparticles (LNPs), polymer particles, and virus-like particles (VLPs) (Table 2). Adjuvants serve as additional vaccine components that enhance the magnitude, breadth, and durability of the immune response to vaccines [103]. Adjuvants, which can vary from synthetic small molecules to complex natural extracts and particulate materials, play a critical role in vaccine development. Selecting the appropriate adjuvant to complement the antigen and enhance immune responses is a key consideration in creating effective new vaccines. The choice of adjuvant can significantly impact the vaccine’s ability to elicit a strong and durable immune response, making it a crucial factor in the development process [104]. Epitope-based subunit vaccines need adjuvant coupling to enhance their immunogenicity [41]. Common adjuvants include synthetic double-stranded RNAs (dsRNAs), glucopyranosyl lipid A (GLA) and derivatives, imidazoquinolines, cytosine-guanine oligodeoxynucleotides (CpG ODNs), cyclic dinucleotides (CDNs), metabolic adjuvants, manganese adjuvants, and derivatives [104]. Cytokine adjuvants are commonly used in vaccine adjuvants, comprising type I interferons (IFN-*α*/*β*/*ω*), FMS-like tyrosine kinase 3 ligand (FLT3-L), interleukin 12 (IL-12), and granulocyte/macrophage-colony stimulating factor (GM-CSF) [105]. Choosing adjuvants based on the specific requirements of different vaccines is essential, and this process often involves extensive experimentation and data analysis to optimize their effectiveness.

## 4. Challenges and Perspective for T-Cell Epitope-Based Vaccines

T-cell epitope-based vaccines offer an innovative approach, particularly for infectious diseases that traditional vaccines, such as inactivated vaccines, struggle to control effectively. These vaccines can induce robust cell-mediated immune responses and demonstrate high conservation and broad applicability in combating emerging diseases. They prove cost-effective and suitable for large-scale vaccination programs, leveraging the multifunctional capabilities and cross-reactivity of T-cells to enhance immune protection. Importantly, they help mitigate the emergence of viral escape mutants [2].

### 4.1. Challenges for Developing T-Cell Epitope-Based Vaccines

The field of epitope-based vaccine development faces several challenges, including identifying suitable antigenic candidate antigens and their dominant immunogenic epitopes, alongside creating an efficient delivery system. Accurate identification of epitopes is crucial for constructing T-cell epitope-based vaccines. Future research should prioritize methods for rapid and precise epitope identification. The genetic diversity of MHC haplotypes represents another significant variable that could influence T-cell epitope-based vaccines [106]. Moreover, factors such as virus mutation, immune escape mechanisms, and strategies to optimize vaccine stability and long-term efficacy must be considered. While software tools have enhanced efficiency and reduced costs in vaccine design, extensive clinical trials remain essential to validate vaccine effectiveness.

Efforts aimed at clarifying the most effective immune mechanisms triggered by vaccination or infection typically concentrate on assessing the correlations of protection [2]. However, these correlates might not fully reveal the true protective mechanisms involved. Additionally, a vaccine might evoke a protective mechanism that differs from the primary effector mechanism observed during natural infection. Furthermore, various immune effector mechanisms that target different viral antigens are likely to work in strong cooperation.

Methods for evaluating cell-mediated immune responses include enzyme-linked immunosorbent assay (ELISA), ELIspot, flow cytometry, single-cell RNA sequencing, qPCR, and immunohistochemistry. Assessing T-cell responses alongside rare clinical outcomes can be complicated and expensive, highlighting the necessity for innovative approaches in clinical trial design for financial efficiency. Current methods have limitations: ELISA detects only cytokines, flow cytometry analyzes cell types and cytokines, and while single-cell RNA sequencing provides insights into single-cell characteristics following flow cytometry, it remains prohibitively expensive. Moreover, technical complexities in T-cell assays have frequently hampered comparative analysis of vaccine-induced cell-mediated immune responses irrespective of humoral responses [106]. Most cell-mediated immune response assays lack standardization across laboratories, limiting inter-study comparisons and complicating accurate assessment of findings across various studies [106]. In the future, it would be beneficial to develop a standardized, highly sensitive test for evaluating cell-mediated immune reactions to ensure uniform data interpretation across studies. Additionally, the assay should establish potential functional relevance. Challenges in measuring cell-mediated immune responses are compounded by the distribution of T-cell subsets and their migration among different sites as the infection evolves.

The transformation of T-cell epitope-based vaccines from the laboratory to clinical trials encounters various challenges, including issues related to safety assessment, effectiveness verification, and large-scale production. Despite these challenges, we firmly believe that the ongoing advancements in AI technologies and the deepening exploration of cell-mediated immune response will increasingly demonstrate the significant advantages of T-cell epitope-based vaccines.

### 4.2. Perspectives for T-Cell Epitope-Based Vaccines

Vaccines will remain a pivotal approach for preventing and controlling infectious diseases. Strategic utilization of key antigenic epitopes with robust immunogenicity will advance the scientific precision of future broad-spectrum and efficient vaccine design improvements. Rational vaccine design based on structural insights holds promise for future vaccine development (Figure 2). For instance, the structure-based RSV vaccine exhibits immune protection against RSV, which is achieved through reasonable mutation modification of the F protein [1,53]. Several vaccines are being evaluated in clinical trials, including M-001 for influenza virus and UB-612 (UBI Asia, Hsinchu, Taiwan, NCT: 04773067) for SARS-CoV-2 (Table 2). Future vaccine strategies must integrate designs that coordinate both cellular and humoral immune responses as this will be critical in enhancing the protective efficacy of new vaccines against infectious diseases [13]. Key principles for epitope-based vaccine design include extensive exploration and utilization of viral protective antigens or epitopes, as well as the development of novel vaccines that integrate protein structure with innovative delivery platforms.

More and more strategies are being employed to predict T-cell epitopes, aiming for high accuracy and low costs in identification processes. Consequently, the development of *in silico* analysis technology is essential for future studies. This entails not only enhancing computing speed but also expanding the inclusion of more clinical models in the database. Additionally, bioinformatics can be utilized to analyze the physicochemical properties and immunogenicity of epitope peptides, significantly reducing the workload of epitope screening. Certainly, it is important to note that there is no foolproof method, and reliance solely on one software or website should be avoided. Instead, a comprehensive analysis that combines multiple methods, along with further *in vitro* and *in vivo* experiments, is necessary to accurately identify epitopes.

## 5. Conclusions

The advantages of T-cell epitope-based vaccines lie in their ability to elicit cell-mediated immune responses, providing long-term protection against viruses and cross-protection against viral variants. Another key benefit is their capacity to target the viral conserved regions, which are relatively resistant to mutation, thereby ensuring protective immune responses when viral variants occur. In summary, T-cell epitopes play a pivotal role in cell-mediated immune responses, and the development of treatment and prevention products based on these epitopes holds immense potential in enhancing our ability to effectively prevent and treat infectious diseases that cannot be controlled by traditional vaccines.

## Figures and Tables

**Table 1:** Different methods for the prediction and analysis of T-cell epitopes.

Methods	Advantages	Disadvantages	Applications
IEDB	Multiple methods, large-scale datasets, and immunogenicity analysis	Accuracy limitations	SARS-CoV-2 [26]
SYFPEITHI	Easy to use, suitable for many MHC molecules	Lack of updated and expanded training data	Human immunodeficiency virus (HIV) [22]
VaxiJen	Immunogenicity and physicochemical properties analysis	Software algorithm is relatively simple	Nipah virus [27]
VirVACPRED	Efficient prediction and processing of multiple sequences	Limited to the prediction of viral proteins	Monkeypox virus [28]
NetCTL	CTL epitopes optimization	Only applicable to MHC-I molecules	Dengue virus (DENV) [29]
NetMHCpan	Highly accurate and suitable for a variety of MHC molecules	Highly computational complexity	ASFV [30], SARS-CoV-2 [26]
NetMHC	Widely used and accuracy	Affected by training data and method selection	HIV [31]
IFNepitope	Predict epitopes that induce IFN-*γ*	Limited datasets	Malaria [32]
MHCflurry	Accurate and applied to a variety of MHC molecules	Requires a lot of training data and computing resources	Oncolytic viruses [33]
EpiMatrix	Optimized	Paid licensing and higher costs	Zaire Ebola virus [34]
MHC2Pred	Accurate, broad-spectrum, and fast	Relying on known data	Epstein-Barr virus [35]
ProPred	Combining multiple methods	Only applicable to MHC-I molecules	Japanese encephalitis virus [36]
RANK-PEP	Multiple species, diverse information	Only applicable to MHC-I molecules	DENV [37]
PS-CPL	High-throughput screening, flexibility, and accuracy	Complexity, high cost, and laboratory limitations	Tumor [38]
CANTiGEN	Integrated database, user-friendly, predictive power	Relying on existing data	Tumor [38]
RANKPEP	Use of experimental data	Lack of comprehensive training data	SARS-CoV-2 [39]
Tepitool	Applicable to both B and T-cell epitopes	Potential gaps in predictive accuracy	SARS-CoV-2 [40]
MHCPred	Predict MHC-I- and -II-binding peptides	Depends on training data quality and algorithms’ ability	SARS-CoV-2 [41]
AlgPred v2.0	Wide range of data and methods	Unknown allergens cannot be predicted	*Mycobacterium tuberculosis* [42]
ToxinPred	Toxicity analysis	Inherent predictive limitations	Human metapneumovirus [43]
